# Western Diet Decreases the Liver Mitochondrial Oxidative Flux of Succinate: Insight from a Murine NAFLD Model

**DOI:** 10.3390/ijms22136908

**Published:** 2021-06-27

**Authors:** Pavla Staňková, Otto Kučera, Eva Peterová, Moustafa Elkalaf, David Rychtrmoc, Jan Melek, Miroslav Podhola, Veronika Zubáňová, Zuzana Červinková

**Affiliations:** 1 Department of Physiology, Faculty of Medicine in Hradec Králové, Charles University, Šimkova 870, 500 03 Hradec Králové, Czech Republic; stankovap@lfhk.cuni.cz (P.S.); peterove@lfhk.cuni.cz (E.P.); elkalafm@lfhk.cuni.cz (M.E.); rychtrmocd@lfhk.cuni.cz (D.R.); jan.melek@lfhk.cuni.cz (J.M.); zubanovv@lfhk.cuni.cz (V.Z.); wolff@lfhk.cuni.cz (Z.Č.); 2 Department of Medical Biochemistry, Faculty of Medicine in Hradec Králové, Charles University, Šimkova 870, 500 03 Hradec Králové, Czech Republic; 3 Department of Pathophysiology, Third Faculty of Medicine, Charles University Prague, Ruská 87, 100 00 Prague, Czech Republic; 4 The Fingerland Department of Pathology, Charles University, Faculty of Medicine in Hradec Králové and University Hospital Hradec Králové, Sokolská 581, 500 05 Hradec Králové, Czech Republic; miroslav.podhola@fnhk.cz; 5 Department of Clinical Biochemistry and Diagnostics, Charles University, Faculty of Medicine in Hradec Králové and University Hospital Hradec Králové, Sokolská 581, 500 05 Hradec Králové, Czech Republic

**Keywords:** nonalcoholic fatty liver disease, mitochondria, oxidative phosphorylation, respirometry, succinate dehydrogenase, succinate

## Abstract

Mitochondria play an essential role in the pathogenesis of nonalcoholic fatty liver disease (NAFLD). Previously, we found that succinate-activated respiration was the most affected mitochondrial parameter in mice with mild NAFLD. In this study, we focused on the role of succinate dehydrogenase (SDH) in NAFLD pathogenesis. To induce the progression of NAFLD to nonalcoholic steatohepatitis (NASH), C57BL/6J mice were fed a Western-style diet (WD) or control diet for 30 weeks. NAFLD severity was evaluated histologically and the expression of selected proteins and genes was assessed. Mitochondrial respiration was measured by high-resolution respirometry. Liver redox status was assessed using glutathione, malondialdehyde, and mitochondrial production of reactive oxygen species (ROS). Metabolomic analysis was performed by GC/MS. WD consumption for 30 weeks led to reduced succinate-activated respiration. We also observed decreased SDH activity, decreased expression of the SDH activator sirtuin 3, decreased gene expression of SDH subunits, and increased levels of hepatic succinate, an important signaling molecule. Succinate receptor 1 (SUCNR1) gene and protein expression were reduced in the livers of WD-fed mice. We did not observe signs of oxidative damage compared to the control group. The changes observed in WD-fed mice appear to be adaptive to prevent mitochondrial respiratory chain overload and massive ROS production.

## 1. Introduction

White adipose tissue (WAT) contributes to systemic metabolic flexibility by efficiently storing surplus fuel in the form of triglycerides (TGs) and by quickly mobilizing fatty acids (FAs) to supply peripheral organs to meet their energetic demands. These processes are coordinated via endocrine cues and metabolic signals [1]. Cellular responses to changes in the nutritional state are predominantly regulated by mitochondria [2]. WAT is highly integrated with other tissues, especially the liver [1]. The liver is a major metabolic organ that coordinates the metabolic flexibility of the whole body, with hepatocyte mitochondria being key partners in fine-tuning this process [3].

The incidence of obesity has been continuously increasing over the last several decades, and its related health complications have become major health problems worldwide [4]. The progression of obesity is associated with the establishment of a low-grade inflammatory status in WAT, leading to multiple pathogenic outcomes, including insulin resistance [5]. The inability to regulate lipolytic and antilipolytic processes in WAT during starvation and feeding leads to metabolic inflexibility [2]. When WAT reaches its maximum capacity or fails to expand, peripheral organs become overwhelmed by energy substrates and fat accumulates in ectopic depots.

NAFLD is recognized as a hepatic manifestation of metabolic syndrome and currently represents the most common chronic liver condition [6]. NAFLD encompasses a wide range of pathologies, including simple steatosis, NASH, fibrosis, and ultimately cirrhosis, which may progress to hepatocellular carcinoma (HCC) [7]. Overloading mitochondria with energy substrates leads to ineffective substrate switching [2]. Competition between substrates and excessive mixed nutrient entry into the electron transfer system (ETS) not matched by energy demand may overload the ETS, resulting in reactive oxygen species (ROS) production [8]. NAFLD is sometimes considered a mitochondrial disease [9]; however, the results from studies on mitochondrial function in NAFLD are contradictory, depending on the stage of the disease, susceptibility of the metabolic pathway, and the ability of hepatocytes to buffer and store excess lipids [10,11]. Whether mitochondrial alterations precede, accompany, or are a consequence of pathogenesis remains a matter of debate [3].

Ethical and legislative reasons limit the possibilities of obtaining human tissues for research purposes, therefore animal models of human diseases are essential tools for the study of pathophysiology and treatment options. WD-fed animal models most closely mimic human NAFLD [12,13,14]. Reproducibility is of crucial importance in any animal model of disease, but in the case of NAFLD, the reproducibility is often low among laboratories. In our previous study, we induced NAFLD in C57BL/6J mice fed a high-fat, high-fructose, and high-cholesterol WD for 24 weeks [14]. WD caused massive liver steatosis accompanied by mild inflammation and fibrosis. Thus, we induced a mild form of NAFLD with a limited stage of fibrosis, which was not suitable for further studies, especially on possible therapeutic intervention in this disease. We observed decreased succinate-activated mitochondrial respiration and decreased activity of SDH, increased ketogenic capacity, but no evidence of oxidative damage in the livers of WD-fed mice. Oxidative flux with other substrates was unaffected. We showed that mitochondria in this stage of the disease were adapted to withstand increased substrate influx [14]. However, inhibition of SDH may lead to the accumulation of succinate, an important signaling molecule associated with hypoxic response, inflammation, fibrosis and carcinogenesis [15,16]. Succinate exerts paracrine and endocrine effects through its widely distributed receptor SUCNR1 [17]. 

An advanced disease model is essential to study pathogenic mechanisms and therapeutic options. The aim of this work was to induce a more severe stage of NAFLD in mice by extending the WD feeding interval to 30 weeks while focusing on succinate metabolism in more detail.

## 2. Results

### 2.1. Morphologic Parameters and Plasma Analysis

Animals fed a 30-week WD (WD30) gained significantly more weight (*p* < 0.01). Their absolute and relative liver weights and the amount of epididymal fat were increased compared to mice fed the control diet for 30 weeks (CD30) (*p* < 0.01) (Table 1). The activities of alanine transaminase (ALT), aspartate transaminase (AST) and alkaline phosphatase (ALP) were elevated in mice fed the WD (*p* < 0.01), as were albumin and total protein concentrations (*p* < 0.01). Levels of blood urea nitrogen (BUN) were decreased compared to control animals (*p* < 0.05) (Table 1).

### 2.2. Epididymal Fat (eWAT) Histology 

**Figure 1:**
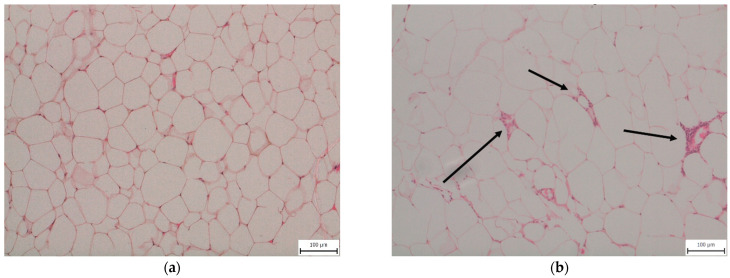
Representative histopathological samples of epididymal adipose tissue taken from mice fed (**a**) a control diet (CD30) or (**b**) a Western-style diet (WD30) for 30 weeks—hematoxylin and eosin staining. Arrows indicate macrophage crown-like structures in the WD30 group.

Feeding animals a 30-week WD induced inflammatory changes in eWAT. We observed macrophage infiltration with the formation of crown-like structures characteristic of stressed insulin-resistant dying adipocytes [13] in the eWAT of all mice fed a WD, while mice fed the CD showed no evidence of eWAT inflammation (Figure 1).

### 2.3. Steatosis, Inflammation, Fibrosis and Apoptosis

**Figure 2:**
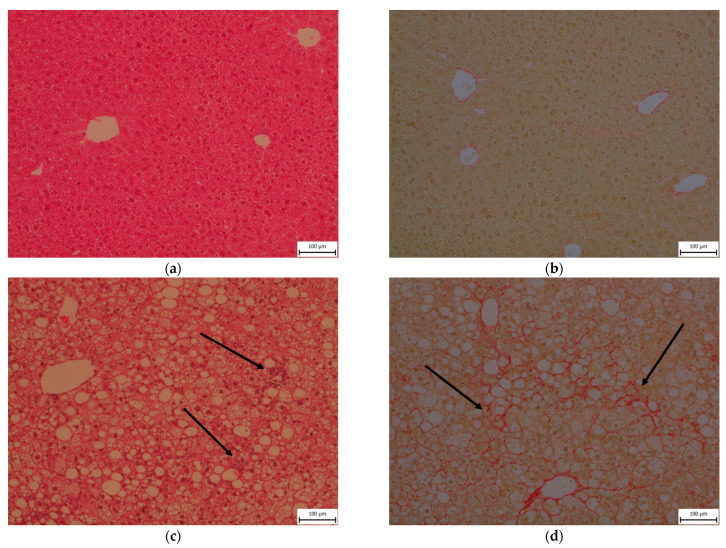
Representative histopathological samples of livers taken from mice fed a control diet (**a**,**b**) or a Western-style diet (**c**,**d**) for 30 weeks. Hematoxylin and eosin staining (**a**,**c**) and sirius red staining (**b**,**d**). Arrows indicate inflammatory infiltrates (**c**) and fibrosis (**d**).

Liver histology was evaluated using the NASH clinical research network scoring system comprising the NAFLD activity score (NAS) (0–8) and fibrosis stage (0–4). The NAS is calculated by summing scores of steatosis (0–3), lobular inflammation (0–3), and hepatocyte ballooning (0–2) [18]. All animals in the WD30 group received a maximum score of 3 for steatosis, which was present in more than 97% of hepatocytes and was equally frequent in both macrovesicular and microvesicular forms. WD feeding also induced lobular inflammation (grade 1–2) and perisinusoidal and portal/periportal fibrosis (stage 2). Although ballooning could not be evaluated, NAS scores of 4 (3 animals) and 5 (3 animals) in the WD30 group indicate a transition from steatosis to NASH [19]. There was no evidence of steatosis, inflammation, or fibrosis in the control group (Figure 2).

Steatosis in the WD30 group was confirmed by increased levels of tissue TGs and cholesterol, and inflammation by elevated levels of liver tumor necrosis factor alpha (TNF-α) and interleukin 6 (IL-6) (*p* < 0.01) (Table 2). Gene expression of the regulators of fibrosis transforming growth factor beta (TGF-β) and tissue inhibitor of metalloproteinases-1 (TIMP-1), and markers of the fibrotic process, collagen type I alpha 2 chain (Col1A2) and alpha smooth muscle actin (α-SMA), were also significantly increased in WD30 group, as well as their protein expression, with the exception of TGF-β and TIMP-1 (Table 2). This could be attributed to the very short half-life of activated TGF-β, which complicates its analysis [20]. The half-life of TIMP-1 protein is also short [21], and its levels are increased only in the livers of NAFLD patients with more advanced fibrosis (stage ≥ 3) [22]. Gene expression of the transcription factor p53 and cyclin-dependent kinase inhibitor 1 (p21) and the apoptotic proteins Bax and Bcl-2 were significantly higher in the WD30 group, as well as their protein expression, with the exception of Bax (Table 2). The plasma membrane channel pannexin 1 (Panx1) contributes to pathophysiological ATP release in lipoapoptosis [23]. WD feeding significantly increased both the gene and protein expression of Panx1 in mouse liver (Table 2).

### 2.4. Mitochondrial Respiration

Citrate synthase (CS) specific activity, frequently used as a functional marker of mitochondria quantity, was not significantly different between the livers of CD30 (80.55 ± 8.72 nmol/min/mg) and WD30 (86.44 ± 14.79 nmol/min/mg) groups.

**Figure 3:**
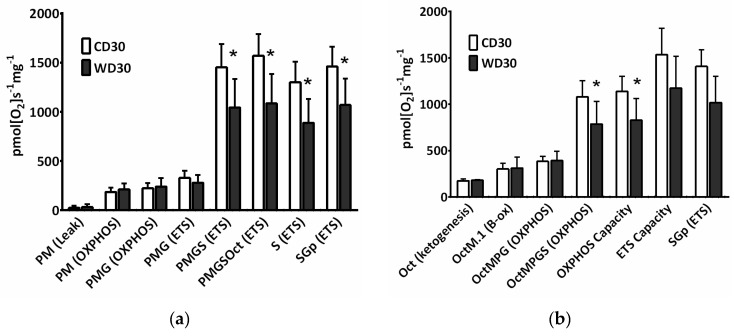
Mitochondrial respiration in mice fed a control diet (CD30) or a Western-style diet (WD30) for 30 weeks. (**a**) Respiratory protocol 1. (**b**) Respiratory protocol 2. Liver homogenates were loaded at a protein concentration of 0.15 mg/mL, and substrates, uncoupler, and inhibitors were gradually added according to the protocol: P (pyruvate, 5 mM), M (malate, 2 mM), M.1 (malate, 0.1 mM), G (glutamate, 10 mM), S (succinate, 50 mM), Oct (octanoylcarnitine, 0.5 mM), and Gp (glycerophosphate, 10 mM). The LEAK state was measured in the presence of reducing substrates but in the absence of ADP. The oxidative phosphorylation (OXPHOS) state was measured in the presence of saturating concentrations of ADP (2.5 mM) and defined reduced substrates. The electron transfer system (ETS) state was measured as oxygen consumption in the noncoupled state at the optimum uncoupler concentration (carbonyl cyanide 4-(trifluoromethoxy)phenylhydrazone, 1.5-2 µM), which was obtained by stepwise titration to induce maximum oxygen flux. Capacity indicates respiration in the presence of all substrates. To inhibit NADH-dependent substrate respiration, rotenone (0.5 µM) was added. Data were corrected for residual oxygen consumption (ROX) as the baseline state. ROX is respiration due to oxidative side reactions that continue after inhibition of the ET pathway (antimycin A, 2.5 µM). Outer mitochondrial membrane integrity was assessed by the addition of cytochrome c (10 µM). Results are expressed as the means ± SD; * *p* < 0.05 (n = 6 each group).

The convergence of different available substrates simultaneously feeding electrons to the ETS must be considered for a better understanding of mitochondrial metabolism. For evaluation of mitochondrial respiration, a mixture of respiratory substrates and substrate-uncoupler-inhibitor titration (SUIT) respiratory protocols (RP) were used [14,24]. We observed decreased oxygen consumption after the addition of succinate (S) in both the oxidative phosphorylation (OXPHOS) and electron transfer system states (ETS) (*p* < 0.05) (Figure 3a,b). When utilizing NADH-dependent substrates (pyruvate P, malate M, glutamate G) in the OXPHOS state, the efficacy of the uncoupler in controlling respiratory flow was slightly but not significantly (*p* = 0.065) reduced. Substrate flux control efficiency is expressed as the percent increase in respiration after substrate addition. Using the RP1, calculated efficiency of the uncoupler was 32.2 ± 6.4% in the CD30 group and 15.5 ± 12.4% in the WD30 group (Figure 3a, PMG (ETS) vs. PMG (OXPHOS)). This may indicate a slight decrease in respiratory reserve capacity for NADH-dependent substrates in the WD30 group. The flux control efficiency for succinate in the OXPHOS state was significantly lower (*p* < 0.01) in the WD30 group (48.9 ± 7%) than in the control group (64.3 ± 2%). Total respiratory reserve capacity was unaffected by WD feeding, although OXPHOS capacity was significantly reduced (*p* < 0.05). We also observed an insignificant reduction in ETS capacity (Figure 3b). 

### 2.5. Succinate Dehydrogenase

**Figure 4:**
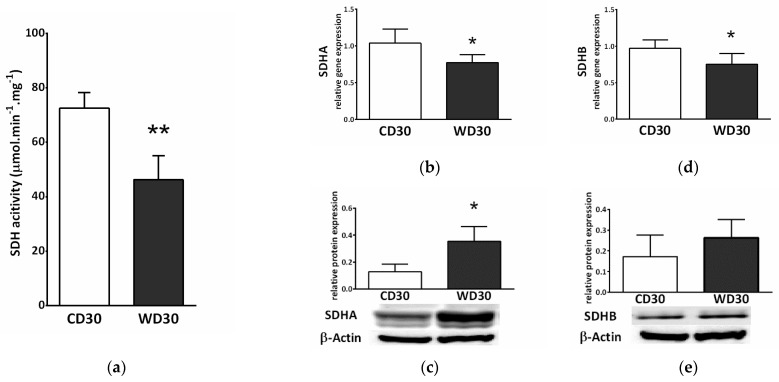
Hepatic succinate dehydrogenase (SDH) in mice fed a control diet (CD30) or a Western-style diet (WD30) for 30 weeks. (**a**) Specific activity of SDH. (**b**) Relative gene expression of SDH subunit A. (**c**) Relative protein expression of SDH subunit A. (**d**) Relative gene expression of SDH subunit B. (**e**) Relative protein expression of SDH subunit B. Results are expressed as the means ± SD; * *p* < 0.05, ** *p* < 0.01 (n = 6 each group).

Feeding mice a WD resulted in a reduction in the specific activity of SDH (*p* < 0.01) (Figure 4a). We also observed decreased gene expression of subunits A and B of SDH (*p* < 0.05) in the WD30 group (Figure 4b,c), although the protein expression of SDHA was significantly increased (*p* < 0.05) (Figure 4c).

**Figure 5:**
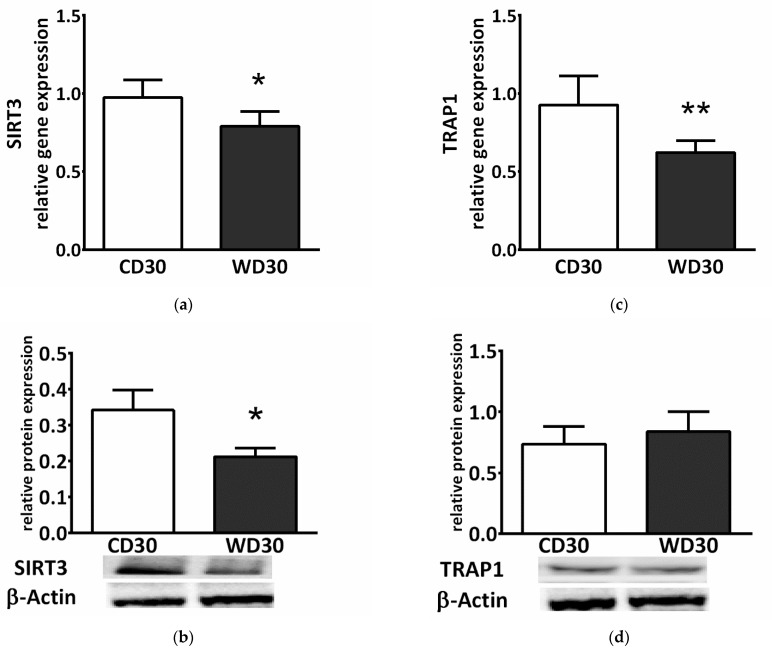
(**a**) Relative hepatic sirtuin 3 (SIRT3) gene and (**b**) protein expression. (**c**) Relative hepatic tumor necrosis factor receptor associated protein 1 (TRAP1) gene and (**d**) protein expression in mice fed a control diet (CD30) or a Western-style diet (WD30) for 30 weeks. Results are expressed as the means ± SD; * *p* < 0.05, ** *p* < 0.01 (n = 6 each group).

SDH activity is highly regulated by posttranslational modifications. We observed decreased gene and protein expression of mitochondrial NAD^+^-dependent deacetylase sirtuin 3 (SIRT3) in the WD30 group (*p* < 0.05) (Figure 5a,b). SDH activity can also be regulated by the mitochondrial chaperone tumor necrosis factor receptor associated protein 1 (TRAP1). WD feeding did not lead to increased TRAP1 protein expression (Figure 5d), and TRAP1 gene expression actually decreased in the WD30 group (*p* < 0.01) (Figure 5c).

### 2.6. Oxidative Stress

**Figure 6:**
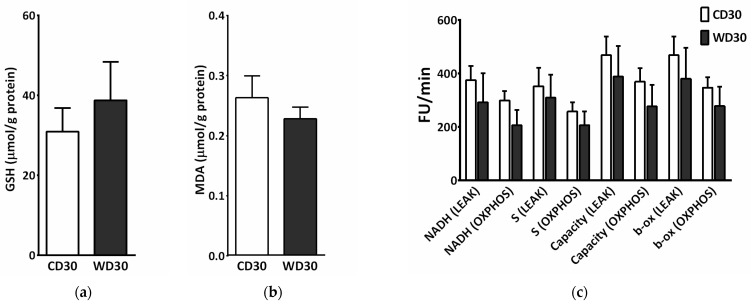
Parameters of liver oxidative stress in mice fed a control diet (CD30) or a Western-style diet (WD30) for 30 weeks. (**a**) Liver reduced glutathione (GSH) level. (**b**) Liver malondialdehyde (MDA) level. (**c**) Production of reactive oxygen species (ROS) in liver homogenate measured as fluorescence changes in dichlorofluorescein. LEAK, measurements in the absence of ADP; OXPHOS, measurements in the presence of ADP (2.5 mM); NADH-linked substrates (pyruvate, 5 mM; glutamate, 10 mM; malate, 2 mM); S (succinate, 10 mM); capacity (NADH-linked substrates + succinate + octanoylcarnitine, 0.5 mM + glycerophosphate, 10 mM); β-oxidation (octanoylcarnitine + malate). Results are expressed as the means ± SD (n = 6 each group).

We did not observe a significant difference between the groups in levels of hepatic reduced glutathione (GSH) or malondialdehyde (MDA) (Figure 6a,b). To measure ROS production, liver homogenates were incubated with a mixture of NADH-linked substrates, succinate, a mixture of NADH- and FADH_2_-linked substrates, and octanoylcarnitine, or octanoylcarnitine plus malate. Measurements were performed in the presence (OXPHOS state) or absence (LEAK state) of adenosine diphosphate (ADP). There was no significant difference in ROS production between groups; however, ROS production appeared to be slightly decreased in the WD30 group (Figure 6c).

**Figure 7:**
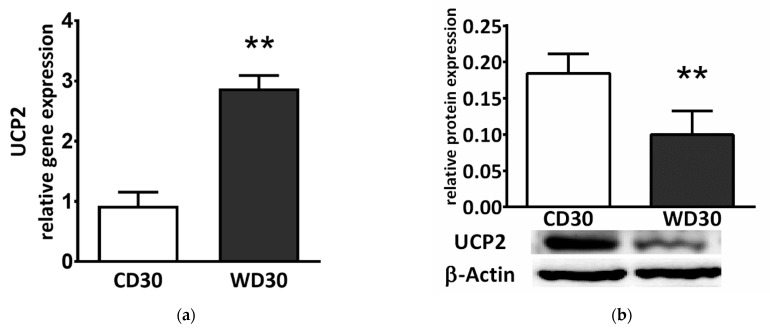
(**a**) Relative hepatic UCP2 gene and (**b**) protein expression in mice fed a control diet (CD30) or a Western-style diet (WD30) for 30 weeks. Results are expressed as the means ± SD; ** *p* < 0.01 (n = 6 each group).

We also determined liver gene and protein expression of uncoupling protein 2 (UCP2). Although gene expression was significantly higher in WD-fed mice (*p* < 0.01) (Figure 7a), protein expression was lower (*p* < 0.01) (Figure 7b).

### 2.7. Metabolomic Analysis

**Figure 8:**
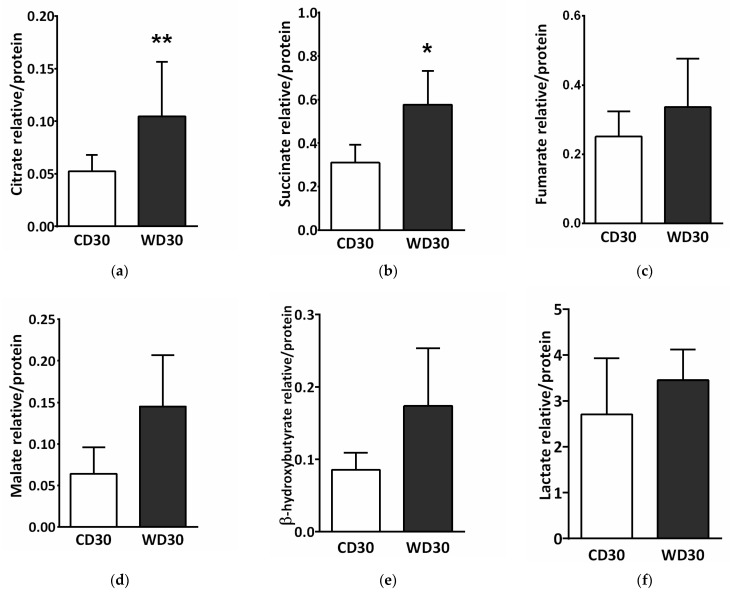
Liver metabolomic analysis in mice fed a control diet (CD30) or a Western-style diet (WD30) for 30 weeks. (**a**) Relative level of citrate. (**b**) Relative level of succinate. (**c**) Relative level of fumarate. (**d**) Relative level of malate. (**e**) Relative level of β-hydroxybutyrate. (**f**) Relative level of lactate. Results are expressed as the means ± SD; * *p* < 0.05, ** *p* < 0.01 (n = 6 each group).

SDH creates the only direct functional link between the tricarboxylic acid (TCA) cycle and oxidative phosphorylation, and is thus optimally situated to adapt and coordinate flux through both pathways [15]. SDH inhibition may lead to the accumulation of succinate and other TCA cycle intermediates involved in the control of cell fate and function [25]. We performed a metabolomics analysis of the liver and determined tissue levels of citrate, succinate, fumarate, malate, β-hydroxybutyrate, and lactate. Levels of all these metabolites were increased in the livers of WD-fed mice (Figure 8), but only changes in citrate (Figure 8a) (*p* < 0.01) and succinate (Figure 8b) (*p* < 0.05) were significant.

### 2.8. Succinate Receptor SUCNR1

**Figure 9:**
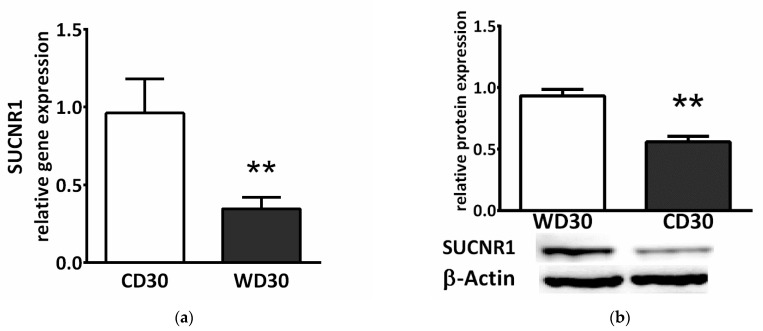
SUCNR1 expression in mice fed a control diet (CD30) or a Western-style diet (WD30) for 30 weeks. (**a**) Relative gene and (**b**) protein expression. Results are expressed as the means ± SD; ** *p* < 0.01 (n = 6 each group).

Succinate exerts a paracrine/endocrine effect through its specific G-protein coupled membrane receptor. We observed decreased relative gene (*p* < 0.01) (Figure 9a) and protein (*p* < 0.01) (Figure 9b) expression of the SUCNR1 receptor in the livers of WD-fed mice.

## 3. Discussion

By extending the WD feeding period from 24 weeks to 30 weeks, we induced a more advanced form of NAFLD in mice. Compared to our previous study [14], we achieved a more profound stage of fibrosis, which was confirmed histologically and by gene and protein expression of fibrogenesis markers. Hepatic TGs (*p* < 0.01) and cholesterol levels (*p* < 0.01) also increased significantly with longer feeding durations.

At the mitochondrial level, 30 weeks of WD administration induced decreased succinate-activated respiration in mice in both the OXPHOS and ETS states. This was also confirmed by the reduced specific activity of SDH in the WD30 group. Compared to the 24-week feeding period, the respiratory flux control efficiency of succinate was significantly reduced (*p* < 0.05). Respiration activated by NADH-dependent substrates was not affected by either length of WD feeding. SDH represents the only direct link between the TCA cycle and the respiratory chain and coordinates the substrate flux in both pathways, indirectly affecting oxidation of NADH-dependent substrates [15]. As previously discussed [14], decreased SDH activity appears to be a general response to chronically increased and dysregulated substrate flux into the mitochondria. 

The catalytic activity of SDH is modulated by posttranslational modifications, as well as active site inhibition by Krebs cycle intermediates and other substances [16,26]. Posttranslational modifications reflect substrate availability, redox status, and energy charge, and participate in fuel selection in the liver [27]. SIRT3, a major NAD^+^-dependent mitochondrial deacetylase regulating SDHA subunit acetylation, increases SDH activity [28] and is its key regulator [29]. SIRT3 activity is modulated by the NAD^+^/NADH ratio, and its expression is decreased in obesity and NAFLD [29,30]. In our NAFLD model, we observed significantly decreased SIRT3 gene and protein expression which is in line with these studies. SDH is also regulated by the mitochondrial molecular chaperone TRAP1, which is induced in most tumor types. TRAP1 displays antioxidant and antiapoptotic activity and is involved in mitochondrial redox control [31]. TRAP1 is able to reprogram metabolism by reducing OXPHOS and promoting glycolysis. TRAP1 overexpression induces fatty liver and plays a critical role in balancing energy substrates during liver regeneration by regulating lipid accumulation [32]. It is highly expressed in the normal liver and has been suggested as a biomarker for HCC, but its physiological role is not clear [33]. It has been found that the antioxidant activity of TRAP1 is mediated by its inhibitory interaction with SDH [34]. To our knowledge, the role of TRAP1 in the pathogenesis of NAFLD has not been evaluated. We assessed protein and gene expression of TRAP1 and found that protein expression was not affected by the WD, while gene expression was actually significantly reduced. This finding excludes the role of TRAP1 in inhibiting SDH at this stage of the disease. TRAP1 expression level probably reflects various cellular contexts, such as the degree of mitochondrial stress [35], and the decrease in TRAP1 gene expression might be a result of efficient adaptation to a chronic excess of energy substrates in the WD30 group, in a manner similar to decreasing TRAP1 expression in cardiomyocytes exposed to hyperglycemic conditions [36]. This may indicate that TRAP1 expression could be differentially affected by energy substrates. 

SDH consists of four nuclear-encoded subunits—two soluble catalytic subunits (SDHA and SDHB) and two integral membrane subunits (SDHC and SDHD) [37]. In addition to reduced SDH activity, we also observed decreased gene expression of the SDHA and SDHB subunits, while protein expression of the SDH subunits was increased. In our previous study, neither gene nor protein expression of the SDHA subunit was significantly affected by the 24-week WD diet [14]. A possible explanation for this discrepancy can be related to the factors involved in assembling the subunits into a functional complex. They mediate the stability of both the individual subunits and the multimeric assembly intermediates [38]. The number of assembly factors identified in this process has been increasing in recent years, illustrating the complexity [39]. The expression of assembly factors may increase under certain stress conditions [40] (e.g., increased succinate levels). Gene expression could be further regulated by proteins through negative feedback [41]. However, these claims are speculative and need to be verified. In this case, the succinate dehydrogenase activity appears to be more influenced by post-translational modifications (SIRT3) and possibly by inhibition by Krebs cycle intermediates. However, the expression of other subunits and assembly factors should be determined, which is beyond the scope of this study.

A high rate of O_2_ consumption stimulated by succinate is linked to the highest rate of ROS production in mammalian mitochondria [42]. In hepatic mitochondria, SDH activity is particularly high due to high flux through cataplerotic and anaplerotic pathways [43]. Although the contribution of SDH to ROS production has long been overlooked, it is now widely accepted that SDH significantly contributes to ROS generation directly and indirectly via reverse electron transfer (RET) through complex I or through matrix NAD^+^ dehydrogenases [37,42,44]. RET is induced by a highly reduced coenzyme Q pool in combination with a large protonmotive force. This occurs in a state of metabolic inflexibility when the substrate overload of mitochondria does not match energy demand [45]. In this case, inhibition of SDH leads to decreased ROS production [46]. We proposed that SDH inhibition is an adaptive mechanism that prevents respiratory chain overload and massive ROS generation [14]. Consistent with this hypothesis, as in the previous study, we did not observe the presence of oxidative damage or increased mitochondrial ROS production in the livers of WD-fed mice compared to control animals, despite the induction of a more advanced form of NAFLD. Other adaptations must also be present, as mitochondrial ROS production was not increased in the WD30 group even by NADH-dependent substrates. We used a mixture of respiratory substrates to measure ROS emissions, and the mitochondria of mice fed the WD could better adapt to this load in comparison to CD30 group, as the loss of metabolic flexibility is associated with oxidation of nutrient mixture regardless of the nutritional context [47]. This may explain the fact that ROS production in the WD30 group was slightly reduced. An initial increase in ROS production may serve as a signaling mechanism to allow upregulation of antioxidant defenses and long-term protection [48]. This is consistent with the slightly elevated levels of reduced glutathione in WD-fed mice. Additionally, a variety of Krebs cycle intermediates have been shown to possess antioxidant properties [49]. Citrate exerts a direct antioxidant effect at a physiological range of concentrations [49]. We observed significantly increased levels of hepatic citrate in mice fed a WD. UCP2 is considered a negative regulator of ROS production through its regulation of mitochondrial membrane potential. The primary physiological functions of UCP2 are redox regulation, ROS handling, and immunity [50]. We observed significantly increased gene expression of UCP2 in the WD30 group; however, its protein expression was significantly decreased. The discrepancy between UCP2 protein and mRNA expression is very common and changes in UCP2 mRNA and protein level are not synonymous [51,52]. UCP2 is regulated at multiple levels including transcription, translation, protein activity, and turnover. Fatty acids enhance UCP2 gene expression through peroxisomal proliferator-activated receptors [53]. The mRNA of UCP2 is constantly suppressed by an upstream open-reading frame [54]. RNA-binding protein heterogeneous nuclear ribonucleoprotein-K and microRNAs (miRNAs) are also involved in the regulation of UCP2 translation [53,55]. For example, the UCP2 transcript is post-transcriptionally downregulated by miR-214 in normal hepatic cells, and miR-214 expression is significantly downregulated in hepatocellular carcinoma patient samples [56]. Moreover, among mitochondrial carriers, the UCP2 protein exhibits an unusually short half-life [57]. Redox state control requires subtle and fast regulation and translational upregulation, which, in combination with the short half-life of UCP2, allows for fast elevation of its protein levels under stressful conditions [53]. It has been proposed that UCP2 can function as a sensor of oxidative stress [58]. The UCP2 mRNA awaiting translation induction represents a functional reserve capacity [59]. Thus, UCP2 does not appear to be directly involved in antioxidant defense at this stage of the disease, but its expression reflects a long-term redox imbalance compensated by adaptation mechanisms. Recently, a second mode of UCP2 influence on redox homeostasis independent of its uncoupling activity has been suggested [53]. UCP2 exports C4 intermediates (aspartate, malate, or oxaloacetate) from the matrix in exchange for the incoming phosphate. UCP2 activity thus decreases the contribution of glucose to mitochondrial oxidative metabolism, promotes oxidation of FAs and glutamine, and stimulates ketogenesis and gluconeogenesis [60]. Thus, UCP2 directly affects the choice of substrates at the mitochondrial level, and its expression can be significantly affected by diet composition, which can explain the differences in expression patterns among studies.

Although oxidative stress is considered an important pathogenic mechanism in the progression of NAFLD, the link between mitochondria-dependent ROS and diet-induced hepatic steatosis is paradoxical and complex [48]. Our findings are consistent with studies by Charlton et al. [12] and Krishnan et al. [61], who did not report increased levels of 8-hydroxydeoxyguanosine or 4-hydroxynonenal, respectively, and whose model we adopted. These results could be affected by the occurrence of a spontaneous mutation in the nicotinamide nucleotide transhydrogenase (NNT) gene of C57BL/6J mice [62]. This mutation may predispose C57BL/6J mice to NASH progression but may also affect the redox state of the control group. CB57BL/6J mice are often used in metabolic studies due to their susceptibility to the development of obesity and insulin resistance. Despite a higher basal ROS production rate in CB57BL/6J mice compared to the CB57BL/6N strain with a functional NNT gene, it was found that oxidative stress was blunted, rather than exacerbated, in the C57BL/6J mice fed a WD for 1 week, likely reflecting compensatory increases in alternative redox buffering pathways [63]. Although the NNT is considered a key antioxidative enzyme, in the heart, reverse-mode NNT is the dominant source of ROS during pressure overload. Due to a mutation of the NNT gene, the C57BL/6J mice are protected from oxidative stress, heart failure, and death [64]. Despite altered redox regulation in CB57BL/6J mice and the absence of signs of oxidative damage in WD-fed mice, NAFLD progression occurred in our study.

Decreased SDH activity leads to the accumulation of succinate and other mitochondrial metabolites involved in controlling chromatin modifications, DNA methylation, and posttranslational modifications of proteins [25]. We observed significantly higher levels of succinate in the livers of WD-fed mice. This is consistent with studies that have reported increased plasma succinate concentrations in a rodent model of NAFLD [65,66]. Elevated plasma succinate concentrations have also been demonstrated in humans with obesity, type 2 diabetes, alcohol-induced liver injury, and NASH [67,68]. In mitochondria, the accumulation of succinate potentially results in the downregulation of gluconeogenesis, aberrations in amino acid catabolism, and protein hypersuccinylation [15,16] Succinate, a competitive inhibitor of α-ketoglutarate-dependent dioxygenases, promotes stabilization of HIF1α, inhibits histone demethylases, and plays an important role in the hypoxic response, metabolic reprogramming during tumorigenesis, and epigenetic modulation [15,16,69]. Outside of cells, succinate exerts para- and endocrine effects through the widespread receptor SUCNR1 (also known as GPR91). Succinate inhibits lipolysis in WAT [28]. As a regulator of inflammation and fibrosis, succinate activates inflammatory and hepatic stellate cells [28,29]. The cross-talk between steatotic hepatocytes and hepatic stellate cells through a unique succinate-SUCNR1 signaling pathway has been postulated to be the molecular basis for NASH-associated hepatic fibrosis [70]. SUCNR1 gene and protein expression was slightly elevated in the livers of C57BL/6J mice fed a WD for 16 weeks, and up-regulated GPR91 receptor expression was evidenced by co-immunohistochemical staining of α-SMA [71]. This seems to contradict our results. We observed significantly reduced gene and protein expression of SUCNR1 in mice fed a WD for 30 weeks. This difference can be partly explained by different durations of WD feeding. In the liver, SUCNR1 is expressed primarily in quiescent stellate cells, and this expression decreases with their activation [72]. Another study revealed that deletion of GPR91 protected mice from obesity induced by a high-fat diet only during the initial period [17]. Moreover, adipose tissue-resident macrophages from obese subjects exhibited decreased expression of SUCNR1 compared to their lean counterparts, and subcutaneous WAT from obese patients was insensitive to signaling through SUCNR1 [73]. SUCNR1 is also highly expressed in immature dendritic cells but is absent in their mature forms [74]. These studies suggest that the activity of SUCNR1 in cells is dynamically regulated and that SUCNR1 may be an early sensor for dietary energy intake. On the other hand, SUCNR1 expression was found to correlate with the severity of fibrosis in human NASH biopsy specimens [71]. SUCNR1 appears to elicit complex contextual and tissue-specific responses and further studies are needed to provide a complete picture of its local and systemic modes of action.

In our previous study, we observed the increased ketogenic capacity of mice fed a WD for 24 weeks compared to control animals, without an increase in FAs oxidation capacity [14]. In this study, the capacity for ketogenesis in mice from the WD30 group was similar to that in the control group, and we observed an insignificant increase in liver β-hydroxybutyrate. Levels of ketone bodies have been reported to be both increased and decreased in individuals with NAFLD [75,76]. The flux through ketogenesis depends on the stage of the disease. At the onset of obesity, it increases and gradually diminishes as the disease progresses [77]. Ketogenesis regulation depends on the utilization of ketones by peripheral tissues and 3-hydroxy-3-methylglutaryl-CoA synthase 2, a key point in the ketogenic pathway, is downregulated by the increased levels of β-hydroxybutyrate. Furthermore, an excess of succinate at the periphery inhibits the utilization of ketone bodies [15]. Ketogenesis could be an ideal pathway for the liver to clear excess energy substrates and is important for regulating the redox state of hepatocytes. Gradual reduction of ketogenic capacity leads to profound hepatocyte TCA cycle overload and might be an important factor in the progression of NAFLD [78].

## 4. Materials and Methods

### 4.1. Animals and Diets

All animals received care according to the guidelines set by the Animal Welfare Body of Charles University (Prague, Czech Republic). Animals were maintained under controlled conditions at 23 ± 1 °C, 55 ± 10% relative humidity, air exchange 12–14 times/h, and 12-h light-dark cycle periods, with free access to food and water. Male C57BL/6J mice (26 ± 2 g, Velaz, Czech Republic) were randomly assigned to one of two groups (n = 6, each group) and fed ad libitum using either a standard control diet (CD, PicoLab RD 20, LabDiet) and tap water (CD30) or a Western- style diet (WD, AIN-76A WD, TestDiet) and glucose (18.1 g/L) and fructose (24 g/L) provided in water over 30 weeks (WD30). Mice were sacrificed under anesthesia, and blood, epididymal fat, and liver samples were harvested for further analysis, kept on ice during processing, or immediately frozen in liquid nitrogen and stored at −80 °C.

### 4.2. Plasma Analysis

Plasma analysis was performed using VetScan Preventive Care Profile Plus and a VS2 chemical analyzer (Canada).

### 4.3. Histology

Fresh samples of liver and epididymal fat samples were fixed in 4% neutral formaldehyde, embedded in paraffin, sectioned and placed on glass slides. Hematoxylin and eosin staining was performed according to standard techniques. Liver fibrosis was quantified using Sirius red staining. Histology was evaluated by a pathologist who was blinded to the dietary condition.

### 4.4. Determination of TGs and Cholesterol

Lipids from the livers were prepared using chloroform-methanol extraction [79]. Total cholesterol and TGs were measured using commercial kits (Roche Diagnostics GmbH, Germany), following the manufacturer’s protocols.

### 4.5. Preparation of Tissue Homogenates and Supernatants

For measurement of mitochondrial respiration, ROS production, determination of GSH, estimation of SDH and CS activities, WB analysis, determination of liver cytokines and TBARS liver homogenates were prepared as described in detail previously [14]. Protein content was evaluated using the Bradford method [80] and the bicinchoninic acid method [81], respectively.

### 4.6. Determination of Liver Cytokines

Concentrations of TNF-α and IL-6 in the supernatant were measured by enzyme-linked immunosorbent assay (BMS622, BMS603, Bender MedSystems, Austria) according to the manufacturer’s instructions.

### 4.7. Western Blot Analysis

Proteins (100 µg) were applied on Novex NuPAGE 4–12% Bis-Tris gel (Invitrogen Life Technologies) under nonreducing conditions and transferred to a 0.2 µm Hybond nitrocellulose membrane (GE Healthcare). Staining with Ponceau S and β-actin were used as loading controls. The membranes were incubated with antibodies against β-Actin (AC-74, Sigma), p21 (F-5, Santa Cruz), p53 (BP53-12, Exbio), BCl-2 (C-2, Santa Cruz), Bax (B-9, Santa Cruz), Timp-1 (D10E6, Cell Signaling), α-SMA (1A4, Sigma), cellular fibronectin (IST-9, Santa Cruz), TGF-β1 (V, Santa Cruz), Col1A (COL-1, Santa Cruz), SDHA (B-1, Sabta Cruz), SDHB (B-1, Santa Cruz), UCP2 (G-6, Santa Cruz), SIRT3 (F-10, Santa Cruz), TRAP-1 (C-8, Santa Cruz), SUNCR1 (GPR91, Abcam), and Pannexin 1 (Abcam) at 4 °C overnight. Secondary antibodies were from Sigma Aldrich. Detection was performed with Western Blotting Luminol Reagent (Santa Cruz) and SuperSignal West Femto (ThermoFisher Scientific). Blots were scanned and quantified using a PXi imaging system (Syngene, Cambridge, UK).

### 4.8. RNA Isolation and Quantitative Polymerase Chain Reaction (qPCR)

Total cellular RNA was extracted using TRIzol reagent (Invitrogen) according to Chomczynski and Sacchi [82]. RNA was reverse transcribed using a cDNA Reverse Transcription Kit and quantified using TaqMan Gene Expression Assays (Acta1 Mm00808218_g1, Fndc3a Mm01312526_m1, TGFb1 Mm03024053_m1, col1a2 Mm01165186_m1, TIMP-1 Mm01341361_m1, Bax Mm01205549_m1, Bcl2 Mm00477631_m1, Cdkn1a Mm00432448_m1, Tp53 Mm01731287_m1, Panx1 Mm01176173_m1, SDHA Mm01352357_m1, SDHB Mm00458272_m1, UCP2 Mm00627599_m1, SIRT3 Mm00452131_m1, TRAP1 Mm00446003_m1, and SUCNR1 Mm00519024_m1). Gene expression was analyzed using a Quantstudio 6 Real-Time PCR system (all obtained from Applied Biosystems, Prague, Czech Republic). Results were normalized to 18S RNA expression. 

### 4.9. Markers of Hepatic Oxidative Stress

Lipoperoxidation was measured by assessing MDA as TBARS [83]. ROS production was monitored using 5-(and 6-)chloromethyl-2’,7´-dichlorodihydrofluorescein diacetate (CM-H2DCFDA, Invitrogen). Liver homogenates (0.1 mg/mL) were incubated in potassium medium (100 mM KCl, 10 mM Tris-HCl, 3 mM MgC_l2_, 4 mM KH_2_PO_4_, 1 mM EDTA, pH 7.4) containing 1 μM CM-H2DCFDA and a mixture of respiratory substrates. The substrates used and their concentrations are shown in the figure legend (Figure 6). The formation of dichlorofluorescein was measured for 1 h at 5-min intervals in a Tecan Infinite M200 (λ_Ex_ = 485 nm, λ_Em_ = 535 nm, 37 °C). For the determination of GSH, liver homogenates were added to cold 5% metaphosphoric acid, shaken, and centrifuged (20,000× *g*, 10 min, 4 °C). Glutathione in the supernatant was analyzed using a modified fluorometric method [84], as previously described [85]. 

### 4.10. Mitochondrial Respiration

Mitochondrial respiration was assessed by high-resolution respirometry (OROBOROS Oxygraph-2k, Austria) with the two SUIT protocols [14]. Measurements were performed in 2 mL of medium MiR05, at 37 °C. The terminology we use for the description of respirometry results is broadly explained in a paper published by COST MitoEAGLE project consortium investigators [24]. Concentrations of substrates, uncoupler, and inhibitors are shown in the legend of Figure 3.

### 4.11. Activity of Succinate Dehydrogenase in Liver Homogenate

SDH activity was measured spectrophotometrically using p-iodonitrotetrazolium as an artificial electron acceptor [86].

### 4.12. Activity of Citrate Synthase in Liver Homogenate

CS activity was measured using a Citrate Synthase Assay Kit (Sigma-Aldrich) according to the manufacturer’s instructions.

### 4.13. Metabolomic Analysis

The derivatizing agent N-tert-butyldimethylsilyl-N-methyltrifluoroacetamide with 1% tert-butyldimethylchlorosilane (TBDMS + 1% TBDMSCl) and anhydrous pyridine were purchased from Sigma Aldrich. N-hexane was purchased from SupraSolv^®^. The internal standard 1-chlorotetradecane (IS) was purchased from laboratory supplies. Approximately 60 mg of liver tissue was taken and immediately frozen with a pair of pliers, precooled in liquid nitrogen, and was placed in cool 70% acetonitrile (0.7 mL/20 mg tissue), followed by homogenization (Ultra-Turrax) on ice. The samples were evenly divided into 3 tubes and centrifuged at 20,000× *g* for 10 min at 4 °C, and 600 μL of supernatant was taken from each tube. One hundred microliters of the mouse liver extract was dried down at laboratory temperature using a SpeedVac (KRD) and reconstituted in 50 μL of anhydrous pyridine. Then, 50 μL of a silylation agent (TBDMS + 1% TBDMSCl) was added. The vial with the reacting mixture was sealed using Teflon tape, vigorously shaken for 10 s and incubated under constant mixing (700 rpm) at 70 °C, for 30 min in a Thermomixer C (Eppendorf). Then, 400 μL hexane was added. Before analysis, 10 μL of an IS (23 μg/mL in hexane) was added and the sample was vigorously shaken for 10 s. Samples were analyzed using two-dimensional comprehensive gas chromatography with mass detection (GCxGC-MS; Pegasus 4D, Leco Corporation, USA) controlled by ChromaTOF v4.5 software. The system was equipped with a robotic injection system (MPS, Gerstel, Germany) controlled by Maestro v1.4. The mass detector was equipped with an EI ion source and a TOF analyzer with unite resolution. A combination of nonpolar and polar separation columns was used for the analyses: primary column: Rxi-5Sil (30 m × 0.25 mm, Restek, Australia); secondary column BPX-50 (1.53 m × 0.1 mm, SGE, Australia). Other parameters were set as follows: inlet temperature 300 °C, injection volume 1 μL in spitless mode, constant He flow 1ml/min, modulation time 3 s (hot pulse 1 s), modulation temperature offset with respect to the secondary oven 15 °C. Temperature program applied to the primary oven: 80 °C (hold 1 min), then increase (15 °C/min) to 320 °C (hold 5 min). The temperature offset applied to the secondary column was +5 °C. Selected compounds were analyzed as tert-butyl silyl derivatives. Analytes were quantified relative to the signal of an IS. Data were processed using ChromaTOF v4.5 software.

### 4.14. Statistical Analysis

Data are expressed as means ± standard deviation (SD). All data processing was performed using GraphPad Prism 6.01 software (La Jolla, CA, USA). Group comparisons were performed using the Mann–Whitney U test. 

## 5. Conclusions

Extending the period of WD feeding from 24 weeks to 30 resulted in the induction of a more advanced stage of NAFLD in C57BL/6J mice. At the mitochondrial level, 30 weeks of WD feeding resulted in decreased succinate-activated respiration, specific SDH activity, and SDHA and SDHB subunit gene expression compared to controls. We also observed increased levels of liver succinate and citrate in WD-fed animals. Succinate is an important signaling molecule and acts through the membrane-bound receptor SUCNR1. In the livers of mice fed a 30-week WD, we observed decreased gene and protein expression of SUCNR1. Given that the SUCNR1 receptor is considered a potential target for NAFLD therapy, more studies are needed to elucidate the regulation of SUCNR1 expression and the complex implications of its activation. Respiration activated by NADH-dependent substrates was not affected by WD feeding, and we observed no signs of oxidative damage in WD-fed C57BL/6J mice compared to control animals. Given that the cell can synthesize only as much ATP as it is able to utilize, the hepatic mitochondrial changes observed in WD-fed mice appear to be adaptations to prevent overload of the respiratory chain rather than an indication of damage.

## Figures and Tables

**Table 1:** Basal phenotype and plasma characteristics in mice fed a control diet (CD30) and Western-style diet (WD30) for 30 weeks.

	Parameter	CD30	WD30
**Phenotype**	Body weight, g	33.0 ± 1.10	47.83 ± 0.75 **
	Absolute liver weight, g	1.51 ± 0.19	4.26 ± 0.50 **
	Relative liver weight, %	4.58 ± 0.62	8.89 ± 0.94 **
	Epididymal fat weight, g	0.75 ± 0.35	2.32 ± 0.35 **
**Plasma biochemistry**	ALT, µkat/L	0.40 ± 0.00	6.65 ± 1.17 **
	AST, µkat/L	0.87 ± 0.13	5.70 ± 0.78 **
	ALP, µkat/L	0.67 ± 0.08	2.27 ± 0.37 **
	BUN, mmol/L	6.30 ± 0.55	5.50 ± 0.33 *
	Albumin, g/L	33.17 ± 3.53	39.00 ± 0.63 **
	Total protein, g/L	48.17 ± 2.20	57.00 ± 2.28 **

The results are expressed as the means ± SD; * *p* < 0.05, ** *p* < 0.01 (n = 6 each group).

**Table 2:** Parameters of liver steatosis, inflammation, fibrosis, and apoptosis in mice fed a control diet (CD30) and Western-style diet (WD30) for 30 weeks.

	Parameter	CD30	WD30
**Steatosis**	TGs, µmol/g tissue	29.79 ± 8.63	169.00 ± 1.98 **
	Cholesterol, µmol/g tissue	6.61 ± 0.52	75.72 ± 12.69 **
**Inflammation**	TNF-α, ng/mg protein	8.83 ± 1.14	21.82 ± 5.19 **
	IL-6, ng/mg protein	17.31 ± 4.30	53.14 ± 12.82 **
**Fibrosis**	TGF-β rel. gene expression	0.645 ± 0.190	1.180 ± 0.124 **
	TGF-β rel. protein expression	0.044 ± 0.072	0.030 ± 0.044
	TIMP-1 rel. gene expression	0.532 ± 0.252	21.29 ± 2.804 **
	TIMP-1 rel. protein expression	0.209± 0.100	0.197± 0.053
	Col1A2 rel. gene expression	0.628 ± 0.250	5.220 ± 0.894 **
	Col1A2 rel. protein expression	17.15 ± 0.2.07	23.51 ± 2.78 **
	α-SMA rel. gene expression	1.667 ± 1.000	5.386 ± 1.872 **
	α-SMA rel. protein expression	0.036 ± 0.006	0.125 ± 0.074 *
**Apoptosis**	Bcl-2 rel. gene expression	0.532 ± 0,252	21.290 ± 2.80 **
	Bcl-2 rel. protein expression	0.097 ± 0.030	0.138 ± 0.024 *
	p53 rel. gene expression	0.725 ± 0.165	0.820 ± 0.053 *
	p53 rel. protein expression	0.070 ± 0.043	0.282 ± 0.103 **
	p21 rel. gene expression	0.606 ± 0.228	6.210 ± 1.468 **
	p21 rel. protein expression	0.391 ± 0.138	0.681 ± 0.188 *
	Bax rel. gene expression	0.913 ± 0.177	1.308 ± 0.154 **
	Bax rel. protein expression	0.184 ± 0.064	0.169 ± 0.028
	Panx1 rel. gene expression	0.693 ± 0.171	1.718 ± 0.207 **
	Panx1 rel protein expression	0.040 ± 0.019	0.089 ± 0.021 **

The results are expressed as the means ± SD; * *p* < 0.05, ** *p* < 0.01 (n = 6 each group).

## Abbreviations

α-SMA	Alpha smooth muscle actin
ADP	Adenosine diphosphate
ALP	Alkaline phosphatase
ALT	Alanine transaminase
AST	Aspartate transaminase
ATP	Adenosine triphosphate
BUN	Blood urea nitrogen
CD	Control diet
CD30	Control diet for 30 weeks
CoA	Coenzyme A
Col1A2	Collagen type I alpha 2 chain
CS	Citrate synthase
ETS	Electron transfer system
eWAT	Epididymal white adipose tissue
FADH_2_	Reduced flavin adenine dinucleotide
FAs	Fatty acids
GSH	Reduced glutathione
HCC	Hepatocellular carcinoma
IL-6	Interleukin 6
LEAK	Respiration measured in the presence of reducing substrates, but the absence of ADP
MDA	Malondialdehyde
NAD^+^	Nicotinamide adenine dinucleotide
NADH	Reduced nicotinamide adenine dinucleotide
NAFLD	Nonalcoholic fatty liver disease
NAS	NAFLD activity score
NASH	Nonalcoholic steatohepatitis
NNT	Nicotinamide nucleotide transhydrogenase
OXPHOS	Oxidative phosphorylation
Panx1	Pannexin 1
p21	Cyclin-dependent kinase inhibitor 1
p53	Tumor protein p53
RET	Reverse electron transfer
ROS	Reactive oxygen species
ROX	Residual oxygen consumption
RP	Respiratory protocol
SD	Standard deviation
SDH	Succinate dehydrogenase
SDHA	Succinate dehydrogenase subunit A
SDHB	Succinate dehydrogenase subunit B
SIRT3	Sirtuin 3
SUCNR1	Succinate receptor
SUIT	Substrate-uncoupler-inhibitor-titration
TBARS	Thiobarbituric acid reactive substances
TCA	Tricarboxylic acid cycle
TGF-β	Transforming growth factor beta
TGs	Triglycerides
TIMP-1	tissue inhibitor of metalloproteinases-1
TNF-α	Tumor necrosis factor alpha
TRAP1	Tumor necrosis factor receptor associated protein 1
UCP2	Uncoupling protein 2
WAT	White adipose tissue
WB	Western blot
WD	Western-style diet
WD30	Western-style diet for 30 weeks
